# Inverted U-shaped relationship between sleep duration and phenotypic age in US adults: a population-based study

**DOI:** 10.1038/s41598-024-56316-7

**Published:** 2024-03-15

**Authors:** Yanwei You, Yuquan Chen, Ruidong Liu, Yangchang Zhang, Meiqing Wang, Zihao Yang, Jianxiu Liu, Xindong Ma

**Affiliations:** 1 Division of Sports Science and Physical Education, Tsinghua University, Beijing, 100084 China; 2 School of Social Sciences, Tsinghua University, Beijing, 100084 China; 3 School of Public Health and Preventive Medicine, Faculty of Medicine, Nursing & Health Sciences, Monash University, Melbourne, VIC 3004 Australia; 4 Sports Coaching College, Beijing Sport University, Beijing, 100091 China; 5 Department of Epidemiology and Biostatistics, School of Public Health, Capital Medical University, Beijing, 100169 China; 6 Vanke School of Public Health, Tsinghua University, Beijing, 100084 China; 7 IDG/McGovern Institute for Brain Research, Tsinghua University, Beijing, 100084 China

**Keywords:** Sleep, Phenotypic age, Exercise, US population, Cross-sectional study, Immunology, Biomarkers, Health care, Risk factors

## Abstract

Sleep is a modifiable behavior that can be targeted in interventions aimed at promoting healthy aging. This study aims to (i) identify the sleep duration trend in US adults; (ii) investigate the relationship between sleep duration and phenotypic age; and (iii) explore the role of exercise in this relationship. Phenotypic age as a novel index was calculated according to biomarkers collected from US adults based on the National Health and Nutrition Examination Survey (NHANES). Sleep information was self-reported by participants and discerned through individual interviews. The principal analytical method employed was weighted multivariable linear regression modeling, which accommodated for the complex multi-stage sampling design. The potential non-linear relationship was explored using a restricted cubic spline (RCS) model. Furthermore, subgroup analyses evaluated the potential effects of sociodemographic and lifestyle factors on the primary study outcomes. A total of 13,569 participants were finally included in, thereby resulting in a weighted population of 78,880,615. An examination of the temporal trends in sleep duration revealed a declining proportion of individuals with insufficient and markedly deficient sleep time since the 2015–2016 cycle. Taken normal sleep group as a reference, participants with extreme short sleep [β (95% CI) 0.582 (0.018, 1.146), *p* = 0.044] and long sleep [β (95% CI) 0.694 (0.186, 1.203), *p* = 0.010] were both positively associated with phenotypic age using the fully adjusted model. According to the dose–response relationship between sleep duration and phenotypic age, long sleep duration can benefit from regular exercise activity, whereas short sleep duration with more exercise tended to have higher phenotypic age. There is an inverted U-shaped relationship between short and long sleep durations and phenotypic age. This study represents an important step forward in our understanding of the complex relationship between sleep and healthy aging. By shedding light on this topic and providing practical exercise recommendations for promoting healthy sleep habits, researchers can help individuals live longer, healthier, and more fulfilling lives.

## Introduction

In today’s fast-paced society, there is a growing trend of people getting insufficient amounts of sleep on a regular basis^1–3^. While the recommended amount of sleep for adults from the National Sleep Foundation is typically between seven and 8 h per night^4,5^, over 1/3 of individuals are falling short of this target due to a variety of factors. There is conflicting evidence regarding sleep duration trends across different countries and regions. While some studies suggest that people are getting less sleep overall, others show no significant change or even an increase in sleep duration^1,6,7^.

While the trends in sleep duration may vary across different countries and regions, there is growing concern about the negative health outcomes associated with chronic sleep health issues^8^. Insufficient sleep duration, defined as less than 7 h per night^4^, has been linked to heightened all-cause mortality risk^9^, obesity^10^, metabolic irregularities^11^, cognitive impairment^12^ and an escalated likelihood of depression^13^. In contrast, a minimum sleep span of at least 7 h per night was found to be correlated with lower estimates of smoking prevalence, physical inactivity and sedentary time, and obesity when compared to shorter sleep durations^14^. Although it seems that patients affected by chronic disorders may tend to exhibit a proclivity towards longer sleep cycles^15^, limited empirical evidence exists to substantiate the contention that extended sleep cycles give rise to untoward health conditions in otherwise healthy adult populations.

In the field of medicine and health, a growing area of interest is the use of “phenotypic age” as a predictor for various diseases and as a biomarker for assessing aging. Phenotypic age refers to an individual’s biological age, which is determined by their physical characteristics and functioning rather than their chronological age^16,17^. Studies have shown that the biological markers based age can be a reliable indicator of an individual's likelihood of developing certain health conditions. This includes chronic diseases such as cardiovascular disease^18^, type 2 diabetes^19^, and neurological disease^20^. One of the advantages of using phenotypic age as a predictive tool is that it can provide more accurate information than chronological age or sole marker (e.g., telomere) alone^21,22^.

Meantime, the influence of sleep on aging is an emerging topic^23,24^ and no consequence has been reached on the relationship between sleep duration and biomarkers-measured aging. Recent studies have suggested that sleep may play a role in telomere length^25^ and thus biological changes during the aging process^26^. One study found that individuals who reported shorter sleep duration had significantly shorter telomeres than those who reported longer sleep durations^27,28^. Another study found that individuals who reported short or long sleep duration had higher levels of Amyloid-β burden, which can contribute to a pathology associated with Alzheimer's disease in its early stages^29^. While these studies provide some evidence for a link between sleep and phenotypic age related changes, more research is needed to fully understand the relationship. Moreover, it is possible that other factors, such as lifestyle habits (e.g., physical activity)^30^, may also play a role in determining an individual’s biological age.

**Figure 1:**
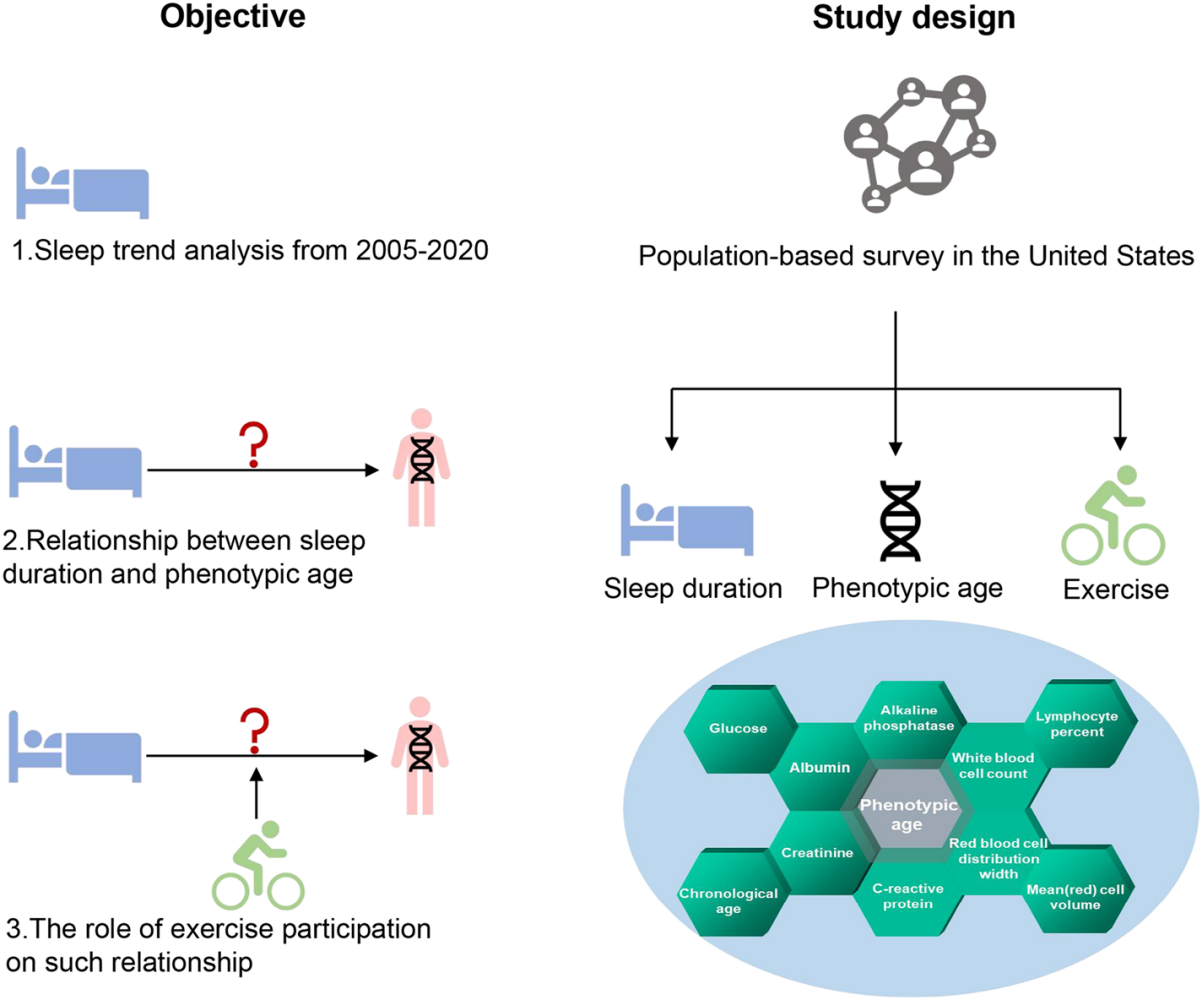
Objective and design of this study.

Based on the aforementioned literature, the burgeoning health problems that are associated with sleep deficiencies demand increased public attention and healthcare resources. Additionally, less is known about the specific relationship between sleep duration and phenotypic age. Therefore, there is an exigency for compelling evidence to awaken public consciousness of the detrimental effects of sleep duration and its influence on aging-based biomarkers. Figure 1 shows the objective and design of the study. By using a nationwide sample of the United States population, this study aims to (i) investigate trends in sleep patterns of US adults from the National Health and Nutrition Examination Survey (NHANES); (ii) evaluate the relationship between sleep and multi-biomarkers-based phenotypic age; (iii) conduct subgroup analysis, and explore whether lifestyle behavior such as exercise participation may impact this relationship.

## Results

**Figure 2:**
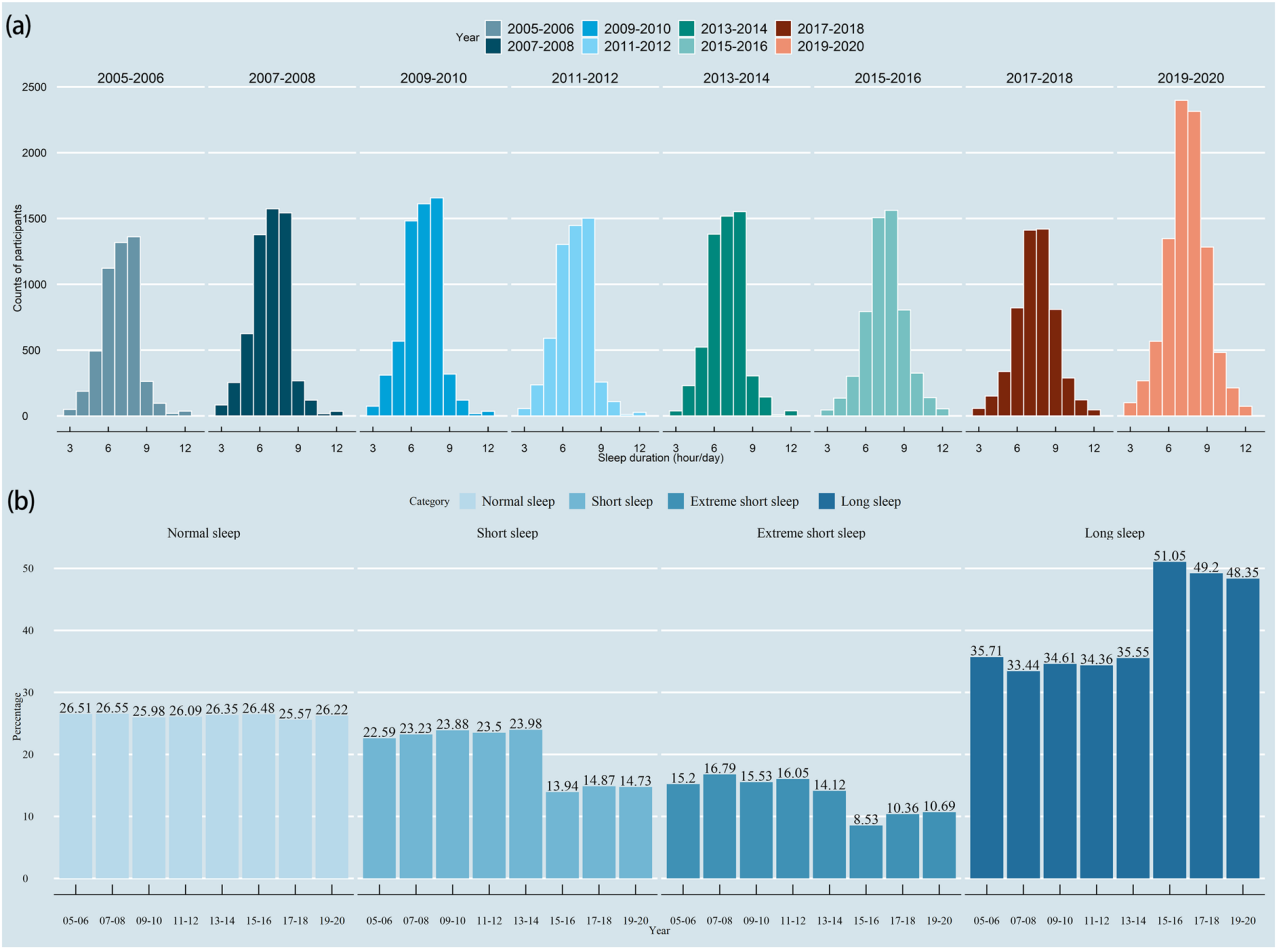
(**a**) Histogram of sleep duration distributions in different year-cycles of NHANES; (**b**) Histogram of year-cycles distributions among different sleep categories.

A total of 48,762 participants from NHANES 2005–2020 were included in the present analysis for detecting the sleep trend. From Fig. 2, it can be found that most people sleep for 6–9 h in different year-cycles. Moreover, the proportion of short sleep and extreme short sleep shows a downward trend, while long sleep duration demonstrates an upward trend since the 2015–2016 cycle. There were 13,569 participants used for the final analysis between sleep duration and phenotypic age, presenting a weighted population of 78,880,615. Table 1 shows the demographic characteristics of the final participants. The sample was uniform across gender (48.88% were males), and most of them were Non-Hispanic Whites (71.64%) and Blacks (10.43%). More than half of them had at least college degree (57.52%) and were married (65.05%). The study participants had an average phenotypic age of 42.76 years.

**Table 1:** Demographic characteristics of the final participants.

Variable	(%/Mean)*	Normal sleep	Short sleep	Extreme short sleep	Long sleep	*p*-value
Chronological age (years)						< 0.001
< 40	36.51	36.34	36.52	36.2	36.78	
[40, 60)	40.01	42.54	42.75	43.81	34.19	
≥ 60	23.49	21.12	20.73	19.99	29.03	
Sex						< 0.001
Male	48.88	50.35	52.44	49.24	44.88	
Female	51.12	49.65	47.56	50.76	55.12	
Race/ethnicity						< 0.001
Non-hispanic White	71.64	76.88	68.3	59.9	73.97	
Non-hispanic Black	10.43	6.44	12.52	20.65	8.44	
Mexican American	7.91	7.34	7.98	6.93	8.8	
Other race/ethnicity	10.02	9.34	11.2	12.52	8.79	
Marital status						< 0.001
Never married	16.29	14.3	16.33	16.54	18	
Married/living with partner	65.05	70.15	64.59	59.4	62.99	
Widowed/divorced	18.66	15.54	19.07	24.06	19.02	
Poverty income ratio						< 0.001
< 1	12.89	9.36	12.55	19.38	13.7	
[1,3)	35.62	32.33	35.86	41.17	36.21	
≥ 3	51.49	58.31	51.59	39.45	50.09	
Education						< 0.001
Below high school	6.14	4.57	5.25	7.46	7.67	
High school	36.34	32.03	38.06	44.44	35.78	
College or above	57.52	63.41	56.7	48.1	56.56	
Body mass index (kg/m^2^)						< 0.001
< 25	31.8	32.88	28.97	26.9	34.8	
[25, 30)	33.5	34.06	33.73	32.16	33.37	
≥ 30	34.7	33.05	37.3	40.94	31.83	
Smokers						< 0.001
Never smoker	52.72	55.67	51.64	45.28	53.82	
Former smoker	24.86	26.06	23.7	21.92	25.78	
Current smoker	22.42	18.27	24.66	32.8	20.41	
Alcohol drinkers						< 0.001
Nondrinker	32.07	28.78	31.23	39.57	32.6	
Moderate alcohol use	47.7	51.84	47.5	39.06	47.57	
High alcohol use	20.23	19.38	21.27	21.37	19.83	
Exercise Activity (min/week)						< 0.001
None	64.85	61.02	64.88	70.35	66.1	
[1, 150)	11.02	12.61	11.05	8.9	10.41	
≥ 150	24.13	26.37	24.07	20.75	23.49	
Hypertension						< 0.001
No	63.9	67.58	63.11	57.86	63.55	
Yes	36.1	32.42	36.89	42.14	36.45	
Diabetes mellitus						< 0.001
No	87.35	89.78	86.73	83.32	87.21	
Yes	12.65	10.22	13.27	16.68	12.79	
Cardiovascular diseases						< 0.001
No	91.56	94.26	92.22	87.59	90.25	
Yes	8.44	5.74	7.78	12.41	9.75	
Phenotypic age (year)	42.76 ± 0.39	41.34 ± 0.45	42.21 ± 0.37	43.77 ± 0.57	44.04 ± 0.58	< 0.001
Red blood cell distribution width (%)	12.75 ± 0.02	12.61 ± 0.02	12.73 ± 0.03	12.99 ± 0.04	12.79 ± 0.02	< 0.001
Mean red cell volume (fL)	89.53 ± 0.16	89.69 ± 0.17	89.35 ± 0.17	89.1 ± 0.22	89.69 ± 0.18	0.003
Lymphocyte percent (%)	30.23 ± 0.13	30 ± 0.17	30.5 ± 0.20	30.54 ± 0.24	30.13 ± 0.16	0.035
White blood cell count (1000 cells/uL)	7.24 ± 0.03	7.09 ± 0.04	7.36 ± 0.06	7.5 ± 0.08	7.19 ± 0.04	< 0.001
Alkaline phosphatase (U/L)	67.72 ± 0.34	65.74 ± 0.47	68.01 ± 0.51	71.25 ± 0.55	67.88 ± 0.47	< 0.001
C-reactive protein (mg/dL)	0.39 ± 0.01	0.34 ± 0.02	0.4 ± 0.02	0.46 ± 0.03	0.41 ± 0.01	< 0.001
Albumin (g/L)	42.66 ± 0.07	42.93 ± 0.08	42.65 ± 0.09	42.15 ± 0.09	42.61 ± 0.1	< 0.001
Glucose (mmol/L)	5.82 ± 0.03	5.75 ± 0.05	5.84 ± 0.05	5.9 ± 0.06	5.83 ± 0.05	0.087
Creatinine (umol/L)	79.38 ± 0.37	79.06 ± 0.49	79.35 ± 0.43	79.96 ± 0.69	79.46 ± 0.55	0.624

*For categorical variables: survey-weighted percentage (%). For continuous variables: survey-weighted mean ± SE; NHANES, National Health and Nutrition Examination Survey.

In the crude model and model 1, sleep duration was found to be not significantly associated with phenotypic age when assessed as a continuous variable, as per Table 2 [Crude Model, β (95% CI) 0.329 (− 0.012, 0.669), *p* = 0.058; Model 1, β (95% CI) − 0.155 (− 0.317, 0.006), *p* = 0.059]. However, in the fully adjusted model, there was a significant association between continuous sleep duration and phenotypic age [Model 2, β (95% CI) 0.153 (0.015, 0.291), *p* = 0.031]. Moreover, this association was held when sleep duration was evaluated as a category variable. When compared to normal sleep group, a positive association between short sleep and phenotypic age was identified in the crude model and model 1 [Crude Model, β (95% CI) 0.867 (0.000, 1.733), *p* = 0.050; Model 1, β (95% CI) 0.837 (0.358, 1.316), *p* < 0.001; Model 2, β (95% CI) 0.142 (− 0.367, 0.650), *p* = 0.570]. Taking normal sleep group as a reference, extreme short sleep was positively associated with phenotypic age [Crude Model, β (95% CI) 2.434 (1.240, 3.628), *p* < 0.001; Model 1, β (95% CI) 2.356 (1.843, 2.869), *p* < 0.001; Model 2, β (95% CI) 0.582 (0.018, 1.146), *p* = 0.044]. Additionally, in comparison to normal sleep group, we also noted a considerably higher phenotypic age in the long sleep group, regardless of all adjusted models [Crude Model, β (95% CI) 2.696 (1.720, 3.672), *p* < 0.001; Model 1, β (95% CI) 1.000 (0.479, 1.521), *p* < 0.001; Model 2, β (95% CI) 0.694 (0.186, 1.203), *p* = 0.010].

**Table 2:** Weighted linear regression results for relationship between sleep duration and phenotypic age.

	Crude model^a^		Model 1^b^		Model 2^c^	
	β (95% CI)	*p*-value	β (95% CI)	*p*-value	β (95% CI)	*p*-value
Sleep duration (hours/day)	0.329 (− 0.012, 0.669)	0.058	− 0.155 (− 0.317, 0.006)	0.059	0.153 (0.015, 0.291)	0.031
Sleep duration (as category)						
Normal sleep	Reference		Reference		Reference	
Short sleep	0.867 (0.000, 1.733)	0.050	0.837 (0.358, 1.316)	< 0.001	0.142 (− 0.367, 0.650)	0.570
Extreme short sleep	2.434 (1.240, 3.628)	< 0.001	2.356 (1.843, 2.869)	< 0.001	0.582 (0.018, 1.146)	0.044
Long sleep	2.696 (1.720, 3.672)	< 0.001	1.000 (0.479, 1.521)	< 0.001	0.694 (0.186, 1.203)	0.010

^a^Crude model, no covariate was adjusted.

^b^Model 1, age, sex, and race were adjusted.

^c^Model 2, age, sex, race, marital status, education, poverty status, body mass index, smokers, alcohol drinkers, exercise activity, hypertension, diabetes mellitus, and cardiovascular diseases were adjusted. CI, confidence interval.

**Figure 3:**
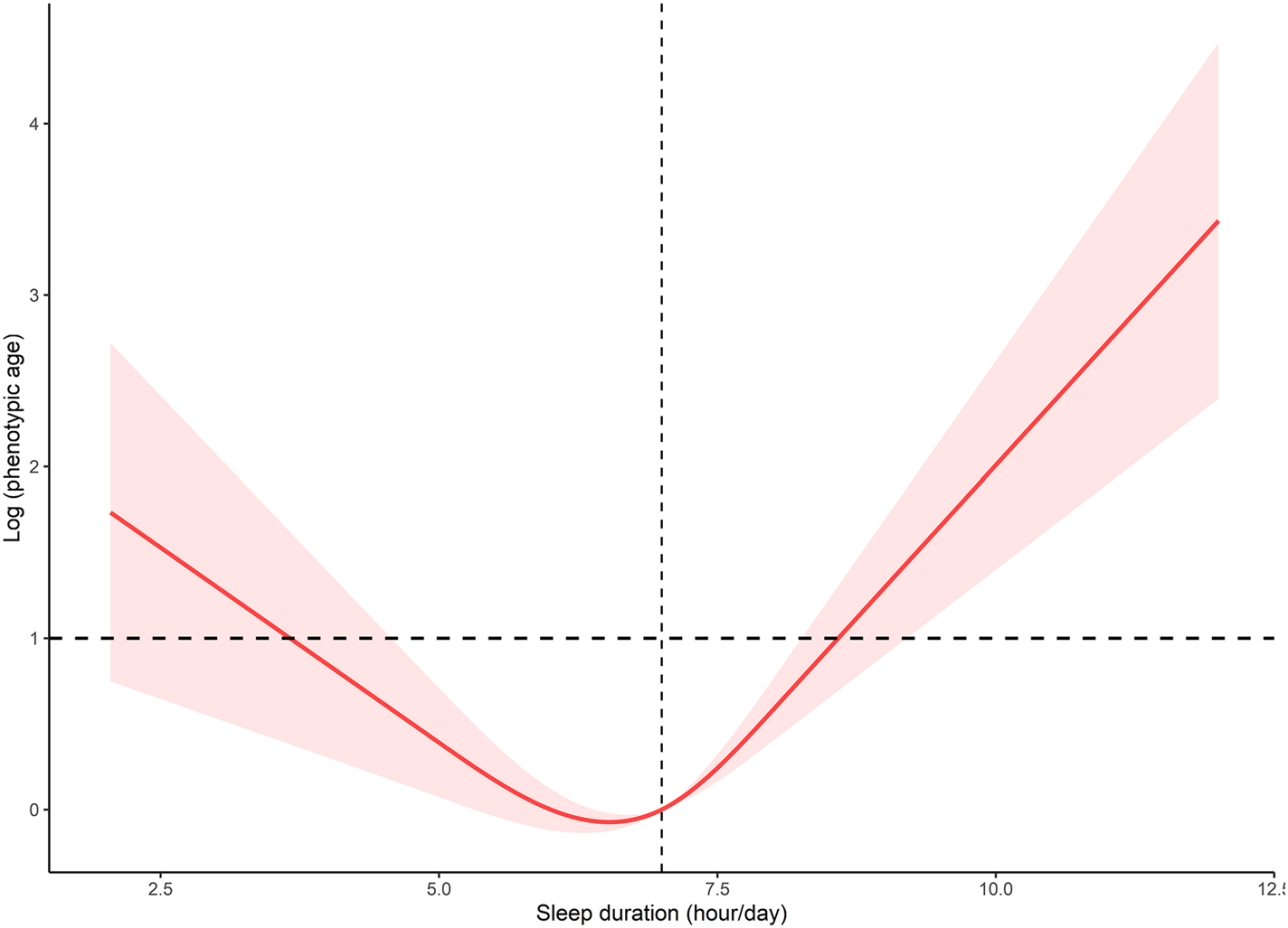
The dose–response relationship between sleep duration and log based phenotypic age.

We calculated the inflection point of the relationship between sleep duration and log based phenotypic age to be 7 h using a two-piecewise linear regression modelling (Table 3). On the left side of the inflection point, the β (95% CI), and *p* value were − 0.010 (− 0.014, − 0.005) and < 0.001, respectively. On the other hand, we observed that there was also a significant association between sleep duration and log based phenotypic age on the right of inflection point [β (95% CI) 0.013 (0.007, 0.018), *p* < 0.001] using the fully adjusted model. Additionally, this dose–response relationship is demonstrated in Fig. 3.

**Table 3:** Threshold effect analysis of relationship between sleep duration and log phenotypic age.

	β (95% CI)	*p*-value
One—line linear regression model	− 0.001 (− 0.003, 0.003)	0.934
Two—piecewise linear regression model		
Sleep duration < 7 (hours/day)	− 0.010 (− 0.014, − 0.005)	< 0.001
Sleep duration ≥ 7 (hours/day)	0.013 (0.007, 0.018)	< 0.001
Log—likelihood ratio test		< 0.001

Age, sex, race, marital status, education, poverty status, body mass index, smokers, alcohol drinkers, exercise activity, hypertension, diabetes mellitus, and cardiovascular diseases were adjusted.

**Figure 4:**
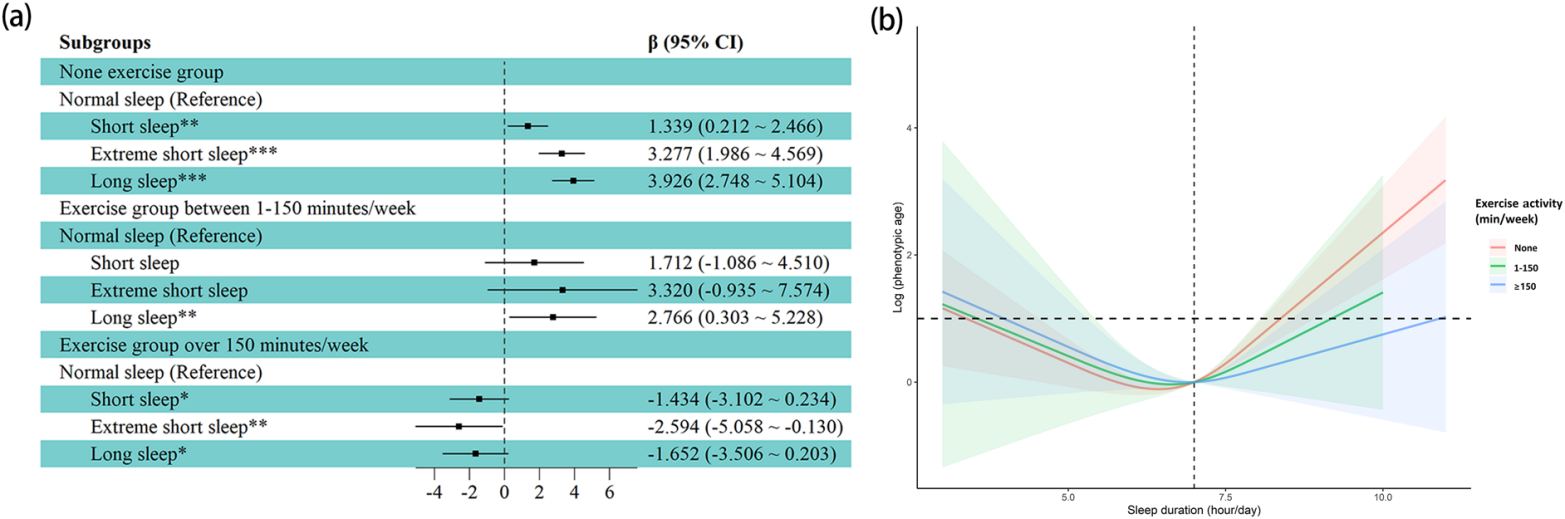
Subgroup analysis (**a**) and dose–response relationship (**b**) between sleep duration and phenotypic age under different level of exercise groups (*p < 0.1, **p < 0.05, ***p < 0.001).

The present study sought to investigate the relationship between sleep duration and phenotypic age by examining the potential influence of demographic, lifestyle and health-related factors. Detailed stratified analyses can be found in Supplementary Table 1. Among these influencing factors, exercise level was a notable variable that also significantly regulated the association mentioned above. Subgroup analysis detected the relationship between sleep duration and phenotypic age under different level of exercise groups (Fig. 4a). Our findings indicated that in none exercise habit group, extreme short sleep and long sleep were positively associated with phenotypic age [short sleep, β (95% CI) 1.339 (0.212, 2.466), *p* = 0.021; extreme short sleep, β (95% CI) 3.277(1.986, 4.569), *p* < 0.001; long sleep, β (95% CI) 3.926(2.748, 5.104), *p* < 0.001]. However, in participants who participated in more than 150 min’ exercise activity per week, there were negative associations between sleep duration and phenotypic age [short sleep, β (95% CI) − 1.434 (− 3.102, 0.234), *p* = 0.089; extreme short sleep, β (95% CI) − 2.594 (− 5.058, − 0.130), *p* = 0.040; long sleep, β (95% CI) − 1.652 (− 3.506, 0.203), *p* = 0.079]. The dose–response relationship between sleep duration and phenotypic age with different exercise activities was further examined using the RCS model. From Fig. 4b, it can be observed that the long sleep duration group can benefit from regular exercise activity, while the short sleep group with more exercise tended to have a higher phenotypic age.

## Discussions

Drawing upon NHANES data, we investigated the sleep duration trend and the relationship between sleep duration and phenotypic age, while also examining the potential effects of confounding factors on such associations. In addition to identifying the relationship, the dose–response and subgroup analysis can provide practical recommendations for promoting healthy sleep habits and slowing down the aging process. Moreover, the results shed light on potential health-related factors such as exercise participation that may influence the relationship between sleep duration and phenotypic age, and have important implications for clinical practice and public health policies.

In the current study, it was observed that extreme short-sleep population demonstrated a downward trend. This dynamic may reflect evolving cultural attitudes toward sleep hygiene, broader societal priorities, as well as demographic shifts. Such findings provide valuable insights into the changing landscape of sleep patterns and attendant health outcomes, thereby informing clinical practice and public health policy. Furthermore, our findings indicated that short sleep was associated with accelerated phenotypic age. Research has shown that getting enough sleep is critical for overall health and wellbeing^31^. Consistent with our findings, it has been proved that insufficient sleep can lead to impaired immune function^32^, which can contribute to an accelerated aging process. In addition, our study also identified that too long sleep was also positively associated with phenotypic age. Numerous investigations have evinced that protracted slumber has been linked with a greater susceptibility to mortality^33,34^. However, this correlation between extended slumber and mortality might be substantially convoluted by variables such as economic standing. Concurrently, surplus activation of catecholaminergic tone and perturbations in energy metabolism were identified as potential drivers behind the correlation between extreme sleep duration and health hazards^34,35^. Findings from different cohort studies further corroborated our results, demonstrating a positive correlation between healthy sleep quality and improved cognitive health, as well as a decreased risk of premature health span decline^36–38^. Additionally, although not explored in this study, the impact of napping on sleep duration is a multifaceted aspect that requires careful consideration^39–41^. Further exploration is needed to understand the relationship between napping habits and overall sleep duration, particularly in the context of split sleep schedules and potential associations with sleep fragmentation.

When it comes to the biological mechanisms about the relationship between sleep duration and hallmarks of aging. It ought to be underscored that critical hormonal modulators implicated in the sleep homeostasis framework, such as serum concentrations of testosterone, were shown to be influenced by inadequate sleep duration and disturbance in circadian rhythms^42^ Furthermore, previous literature posited that an escalation in inflammatory processes could serve as a plausible intermediary mechanism responsible for the augmented aging observed in abnormal sleep^43,44^. It has been reported that transitory deficiency in sleep duration precipitates a reduction in the levels of circulating metabolites orchestrating redox homeostasis, and induces alterations in epigenetic profiles, thereby triggering multifarious downstream effects on biological function^45,46^. In addition, the accelerated aging associated with extreme sleep duration can be interpreted by cellular senescence, which can be reflected by changes in telomere length^47^.

The multifactorial nature of phenotypic age engenders a complex interplay of influential lifestyle factors. Physical activity represents a lifestyle intervention capable of engendering salutary effects on the trajectory of aging and conferring longevity upon its ardent practitioners^48,49^. Our investigation has probed the possibility of interventions to mitigate phenotypic aging and has underscored the salutary role of physical exercise as a lifestyle intervention, particularly in the context of the relationship between sleep and phenotypic age. The import of our research lies in its emphasis on the capacity of physical exercise to bestow benefits upon individuals with over 7 h of nightly slumber. It is noteworthy that a promising alternative therapeutic avenue for mitigating sleep disturbances in individuals across the lifespan, spanning from young to geriatric populations, has emerged in the form of exercise^50–52^. According to our results, individuals with reduced sleep duration may experience accelerated phenotypic aging despite more regular engagement in exercise regimens. At a glance, the present findings may appear antithetical to the well-documented benefits of exercise. However, in consideration of the fact that short sleep itself could affect the ability and motivation of exercise, it makes sense that exercise intensity could be an important factor. There is current evidence finding that the benefits of exercise on health may have a threshold effect on both young and older adults^53,54^. From mechanism, conducting one- bout high volume of exercise might increase the inflammatory response^55^, especially considering the short-sleep status. Hence, the premise of positive benefits of exercise is regular circadian rhythm and sufficient sleep.

Our study has several strengths. The importance of large-scale studies of sleep should be recognized^56^. First, we used data from a large, nationally representative sample of US adults, increasing the generalizability of our findings. Second, we utilized the concept of phenotypic age, which provides a more comprehensive measure of biological aging than chronological age alone. Third, we took into account various sociodemographic and health-related factors that could confound the relationship between sleep duration and phenotypic age. Moreover, another key aspect of this study was its focus on the influence of physical exercise. By examining how different subgroups of exercise groups may impact the relationship between sleep and aging, this study identified that biological aging can be mitigated by sufficient sleep accompanied by regular exercise volume.

Despite these strengths, our study also has some limitations. Firstly, we used cross-sectional data, which limited our ability to establish causality between sleep duration and phenotypic age. The cross-sectional nature of the study design was unable for us to observe the dynamic physiological changes in phenotypic age. Secondly, there were also other influencing factors such as race, sex, age, and BMI, that may modify the observed relationships between sleep duration, exercise, and phenotypic age, which can be further explored. Thirdly, our study relied on self-reported measures of sleep duration (cannot measure time in bed and total sleep time simultaneously), which may be subject to recall bias. Furthermore, most epidemiological studies that rely on self-reported sleep duration (without all-night EEG sleep recordings) suffer from another limitation, namely they cannot determine whether sleep fragmentation, with or without changes in sleep duration, can also affect life span. Fourthly, there is an absence of detailed information on sleep medication usage in the NHANES dataset. Consequently, our analysis did not encompass an evaluation of the potential impact of sleep medication on the relationship between sleep duration and phenotypic age. Finally, we did not explore the role of sleep quality and sleep variability, which may be an important factor in the relationship between sleep duration and phenotypic age. Future studies should consider using objective measures of sleep, such as an actigraphy or polysomnography.

## Conclusions

Overall, the findings of our study suggested that in the United States, the population with extremely short sleep duration showed a decreasing trend in recent years. Moreover, there existed an inverted U-shaped relationship between sleep duration and phenotypic age. This study was significant as it contributed to the growing body of research that emphasized the importance of sleep in relation to biological aging. Additionally, in individuals with extended sleep duration, consistent engagement in regular exercise is associated with benefits, while those with shorter sleep duration and increased exercise exhibit a tendency toward higher phenotypic age. These findings have important implications for public health, underscoring the need for interventions aimed at promoting healthy sleep habits for fostering healthy aging. Further research is needed to establish causality and explore the role of sleep quality in this relationship.

## Methods

### Study population

Study participants are from the NHANES, a comprehensive population-based survey with the aim of collecting data from the civilian population in the United States. As part of NHANES, approximately 10,000 people were surveyed on a 2-year cycle and a multistage probability sampling approach was used to select a sample representative of noninstitutionalized households.

**Figure 5:**
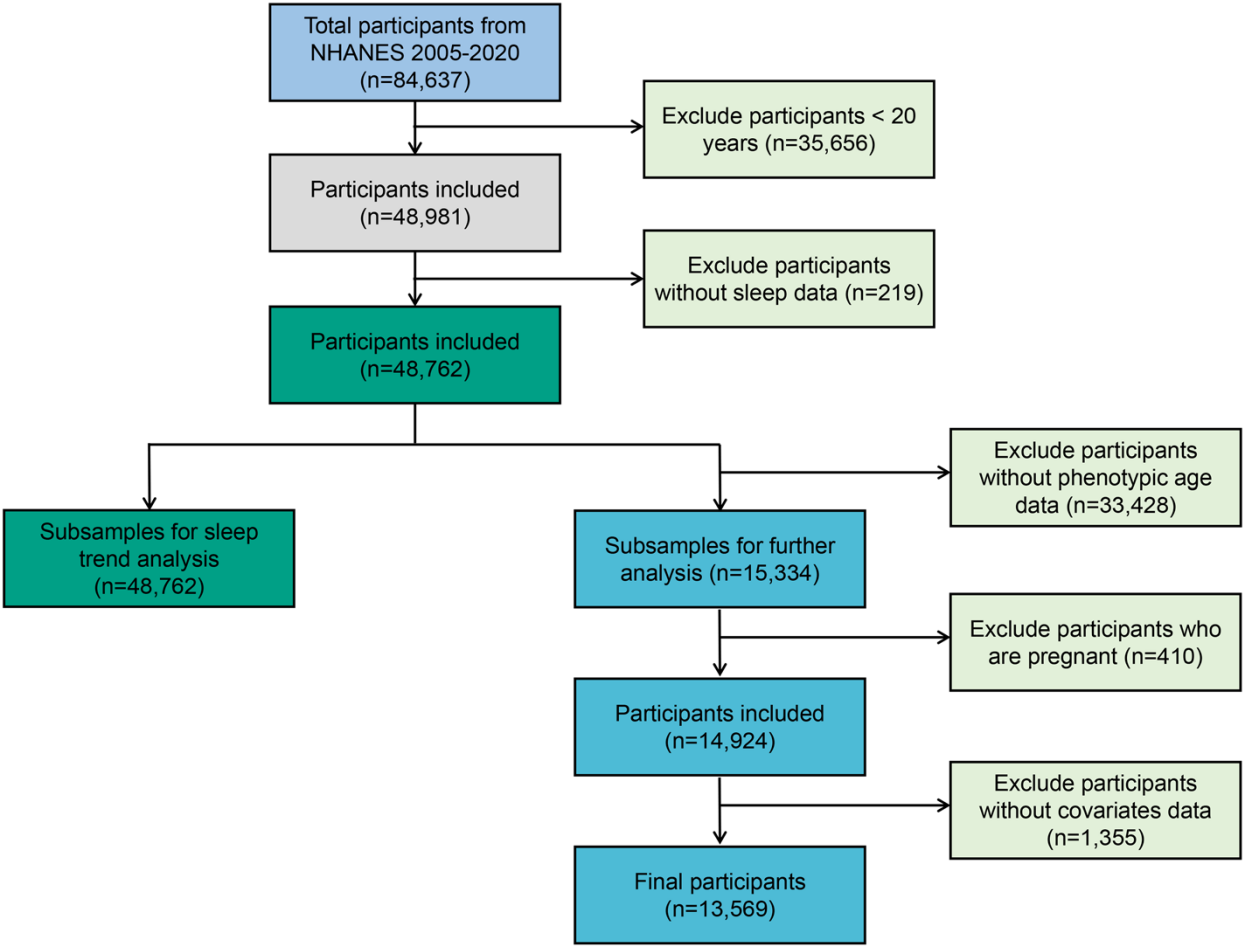
Flowchart of the study design and participants’ inclusion criteria.

In the present study, for the sleep trend analysis, we analyzed participants from eight cycles of the “continuous NHANES” (2005–2020) and included 48,762 participants in the analysis. Considering that the data for phenotypic age was only available in NHANES 2001–2010, a total of 13,569 participants were used for the association between sleep duration and phenotypic age. Participants without sleep data, phenotypic age, and covariates and those who were pregnant were excluded from the analysis. A flowchart showing the inclusion and exclusion process is shown in Fig. 5.

### Measurement of exposure and outcome variables

The exposure variable in this study was sleep duration. NHANES collected self-reported sleep duration data through their standardized questionnaire, which is administered to participants during in-person interviews^57^. In the survey, participants were asked, "How much sleep (hours) do you usually get during the weekdays or during workdays at night? Participants replied with a value between 3 and 12, and responses less than 3 h or more than 12 h were coded as 3 and 12, respectively^58^. Referring to the suggestions by the National Sleep Foundation^5^, sleep duration was categorized into long (≥ 8 h), normal (≥ 7 and < 8 h), short (≥ 6 and < 7 h) and extreme short (< 6 h) sleep.

The outcome variable was phenotypic age. It is apparent that the utilization of a newly fashioned phenotypic age, in lieu of relying on the self-sufficient chronological age, yields superior prognostic outcomes pertaining to health. To be precise, referring to the definition of phenotypic age proposed by Morgan E. Levine et al.^17^, we conducted a computation of the phenotypic age using ten age-linked variables. These include chronological age, albumin (liver), creatinine (kidney), glucose (metabolic), C-reactive protein (inflammation), lymphocyte percent (immune), mean red cell volume (immune), red blood cell distribution width (immune), alkaline phosphatase (liver), and white blood cell count (immune). Participants were required to fast for at least 8 h before giving blood samples. The blood samples were collected at the mobile examination center using standard procedures and stored in a secure facility^59,60^. The method of calculation for phenotypic age was conducted as follows, which have been documented in an existing literature^61^:

$$ {\text{Phenotypic age}} = 141.50{ } + { }\frac{{Ln\left[ { - 0.00553 \times {\text{Ln}}\left( {\exp \left( { \frac{{ - 1.51714 \times \exp \left( {xb} \right)}}{0.0076927}} \right)} \right)} \right]}}{0.09165} $$

### Covariate assessment

The variables that were deemed confounding factors were age groups [< 40, 40–60 (≥ 40 and < 60), or ≥ 60 years], sex groups (male or female), race or ethnicity groups (Non-Hispanic white, Non-Hispanic black, Mexican–American, or other), marital groups (Never married, married/living with partner, widowed/divorced), poverty income ratio groups [< 1,1–3 (≥ 1 and < 3), ≥ 3], education level groups (below high school, high school, college or above), body mass index groups [< 25, 25–30 (≥ 25 and < 30), ≥ 30 kg/m^2^], smoke group (never smoker, former smoker, current smoker), alcohol drink group (nondrinker, moderate alcohol use, high alcohol use), exercise group [none (< 1), 1–150 (≥ 1 and < 150), ≥ 150 min/week], hypertension (yes or no), diabetes mellitus (yes or no), and cardiovascular diseases (yes or no). Individuals classified as moderate alcohol use consumed 14 or fewer drinks per week for men, or 7 or fewer drinks per week for women, with no more than 5 drinks on any single day in the past year. On the other hand, high alcohol use participants were those who consumed more than 14 drinks per week for men, or more than 7 drinks per week for women, including having 5 or more drinks on at least 1 day in the past year for both men and women^62,63^. Exercise, as distinct from work-related physical activities (which included chores, yard work, and other paid or unpaid work), was defined as leisure-time physical engagement, including sports, fitness, and other leisure pursuits. The category of exercise level was suggested by WHO Guidelines and previous literature^64,65^. Detailed selection and classification of covariates can be found in previous publications^64,66^.

### Statistical analyses

All data were combined according to the NHANES protocol, and data analysis was applied using the weighting methodology by the NHANES survey-weighted analytic suggestions. Weights from the Mobile Examination Center (MEC) interviews were reweighted to account for non-responders, non-coverage, and unequal probability of selection in NHANES. For the baseline characteristics of participants, in order to explicate the findings, the continuous variables were articulated as means and standard error (SE), while the categorical variables were articulated as percentages (%). Employing a weighted linear regression model, we investigated the association between sleep duration and phenotypic age, accounting for several confounding variables across three distinct models. The Crude model allowed no adjustment for covariates, whereas Model 1 adjusted for age, sex, and race. In contrast, Model 2 integrated additional covariates including marital status, education, poverty status, body mass index, exercise activity, smokers, alcohol drinkers, hypertension, diabetes mellitus, and cardiovascular diseases to obtain a more accurate estimation of the strength and direction of the relationship under scrutiny.

Furthermore, the dose–response relationship was examined using the threshold effect analysis. Initially, the employment of a smooth curve fitting technique is implemented as a preliminary analysis to discern whether the independent variable has been partitioned into discrete intervals. Then, segmented regression, also referred to as piece-wise regression, is employed whereby separate line segments are utilized to fit each interval. A log-likelihood ratio test is employed in order to compare the one-line (non-segmented) model with the segmented regression model to determine whether a threshold exists. Subsequently, the inflection point connecting the segments that maximizes the likelihood based on the model is determined using a two-step recursive method. More details about the inflection point calculation can be found elsewhere^67^. Upon identification of the inflection point, the nonlinear association was assessed using the restricted cubic spline (RCS) with optimal knots set at three. Logarithmic transformations with natural log were applied to phenotypic age to better reflect changing trends in RCS analysis. Moreover, stratified analyses were performed to investigate the impact of lifestyle factors on the correlation between sleep duration and phenotypic age. Statistical analyses were conducted utilizing the software provided by the R Foundation (accessible via http://www.R-project.org), with statistical significance set at a *p* value of 0.05 or lower.

### Ethics approval

The authors are accountable for all aspects of the work in ensuring that questions related to the accuracy or integrity of any part of the work are appropriately investigated and resolved. The study was conducted in accordance with the Declaration of Helsinki. All information from the NHANES program is available and free for public, so the agreement of the medical ethics committee board was not necessary.

### Consent to participate

Informed consent was obtained from all individual participants included in the study.

### Supplementary Information

Supplementary Table S1.

## Data Availability

The data that support the findings of this study are openly available in https://www.cdc.gov/nchs/nhanes/. Information from NHANES is made available through an extensive series of publications and articles in scientific and technical journals. The original contributions presented in the study were included in the article, further inquiries can be directed to the corresponding author.
