# Supplementary material for: Inverted U-shaped relationship between sleep duration and phenotypic age in US adults: a population-based study
Source: Sci Rep. 2024 Mar 15;14:6247. doi: 10.1038/s41598-024-56316-7 (PMC10940593; doi:10.1038/s41598-024-56316-7)
Supplement: Supplementary file 1 — Supplementary Table S1. [file 41598_2024_56316_MOESM1_ESM.docx]

Table S1. Weighted linear regression of stratified results for associations between sleep duration and phenotypic age.

|  | Normal sleep | Short sleep | *p* | Extreme short sleep | *p* | Long sleep | *p* |
| --- | --- | --- | --- | --- | --- | --- | --- |
| Age |  |  |  |  |  |  |  |
| < 40 | Reference | 0.96( 0.287,1.634) | 0.006 | 2.563( 1.616,3.509) | <0.001 | -0.229(-0.876,0.417) | 0.479 |
| [40, 60) | Reference | 0.926(-0.053,1.905) | 0.063 | 2.242( 1.273,3.210) | <0.001 | 0.567(-0.264,1.399) | 0.176 |
| ≥ 60 | Reference | 1.143(0.054,2.232) | 0.040 | 3.669(2.387,4.950) | <0.001 | 3.419(2.322,4.516) | <0.001 |
| Sex |  |  |  |  |  |  |  |
| Male | Reference | 0.615(-0.709,1.940) | 0.354 | 1.931( 0.314,3.548) | 0.020 | 3.179( 1.725,4.633) | <0.001 |
| Female | Reference | 1.158(-0.258,2.574) | 0.106 | 2.915( 1.406,4.423) | <0.001 | 2.27( 0.958,3.582) | 0.001 |
| Race/ethnicity |  |  |  |  |  |  |  |
| Non-hispanic White | Reference | 0.954(-0.195,2.102) | 0.101 | 2.077( 0.527,3.626) | 0.010 | 3.321( 2.194,4.447) | <0.001 |
| Non-hispanic Black | Reference | 1.346(-0.654,3.346) | 0.181 | 2.91( 0.412,5.407) | 0.024 | 3.338( 0.086,6.589) | 0.044 |
| Mexican American | Reference | 1.885( 0.003,3.767) | 0.050 | 4.849( 2.208,7.489) | <0.001 | 0.422(-1.800,2.643) | 0.704 |
| Other race/ethnicity | Reference | 1.444(-1.040,3.928) | 0.247 | 5.262( 2.557,7.967) | <0.001 | 0.327(-2.800,3.453) | 0.834 |
| Marital status |  |  |  |  |  |  |  |
| Never married | Reference | 2.963( 1.216,4.709) | 0.001 | 4.837( 2.125,7.548) | <0.001 | 0.78(-0.940,2.501) | 0.366 |
| Married/living with partner | Reference | 0.382(-0.714,1.477) | 0.486 | 2.587( 1.158,4.015) | <0.001 | 3.029( 2.065,3.994) | <0.001 |
| Widowed/ divorced | Reference | 0.278(-2.119,2.675) | 0.816 | -2.15(-4.863,0.563) | 0.117 | 4.366( 2.087,6.645) | <0.001 |
| Poverty income ratio |  |  |  |  |  |  |  |
| < 1 | Reference | 1.834(-0.309,3.978) | 0.092 | 6.94( 4.452,9.429) | <0.0001 | 1.609(-0.814,4.033) | 0.188 |
| [1,3) | Reference | 1.125(-0.525,2.774) | 0.176 | 2.748( 0.755,4.741) | 0.008 | 4.648( 2.806,6.490) | <0.001 |
| ≥ 3 | Reference | 0.718(-0.593,2.029) | 0.275 | 1.038(-0.582,2.658) | 0.203 | 1.971( 0.582,3.360) | 0.006 |
| Education |  |  |  |  |  |  |  |
| Below high school | Reference | 3.341(-0.187, 6.868) | 0.063 | 8.77( 4.043,13.498) | <0.001 | 5.677( 1.629, 9.724) | 0.007 |
| High school | Reference | 0.042(-1.836,1.921) | 0.964 | 1.275(-0.620,3.169) | 0.182 | 2.763( 1.226,4.299) | <0.001 |
| College or above | Reference | 0.729(-0.489,1.947) | 0.234 | 1.184(-0.272,2.640) | 0.108 | 1.628( 0.302,2.954) | 0.017 |
| BMI (kg/m^2^) |  |  |  |  |  |  |  |
| < 25 | Reference | 0.714(-1.424,2.853) | 0.504 | 3.394( 1.302,5.486) | 0.002 | 1.887( 0.279,3.495) | 0.022 |
| [25, 30) | Reference | 0.445(-1.107,1.998) | 0.566 | 1.002(-0.661,2.664) | 0.231 | 2.737( 1.195,4.278) | <0.001 |
| ≥ 30 | Reference | 0.462(-1.216,2.141) | 0.582 | 1.587( 0.060,3.114) | 0.042 | 3.99( 2.622,5.359) | <0.001 |
| Smokers |  |  |  |  |  |  |  |
| Never smoker | Reference | 1.522(0.299,2.745) | 0.016 | 3.542(1.330,5.755) | 0.002 | 2.34(1.033,3.647) | <0.001 |
| Former smoker | Reference | 1.431(-0.768,3.629) | 0.196 | 4.059( 1.432,6.687) | 0.003 | 6.479( 4.280,8.678) | <0.001 |
| Current smoker | Reference | -0.349(-2.633,1.936) | 0.760 | 0.74(-1.334,2.813) | 0.476 | -1.046(-2.808,0.715) | 0.238 |
| Alcohol drinkers |  |  |  |  |  |  |  |
| Nondrinker | Reference | 1.702(0.392,3.011) | 0.012 | 3.863(2.104,5.623) | <0.001 | 4.876(3.317,6.435) | <0.001 |
| Moderate alcohol use | Reference | 0.859(-0.554,2.272) | 0.227 | 0.601(-1.190,2.391) | 0.502 | 1.474( 0.065,2.882) | 0.041 |
| High alcohol use | Reference | 0.248(-1.976,2.472) | 0.823 | 2.665( 0.668,4.661) | 0.010 | 1.73(-0.021,3.481) | 0.053 |
| Exercise Activity (min/week) |  |  |  |  |  |  |  |
| None | Reference | 1.339(0.212,2.466) | 0.021 | 3.277(1.986,4.569) | <0.001 | 3.926(2.748,5.104) | <0.001 |
| [1, 150) | Reference | 1.712(-1.086,4.510) | 0.221 | 3.32(-0.935,7.574) | 0.121 | 2.766( 0.303,5.228) | 0.029 |
| ≥ 150 | Reference | -1.434(-3.102, 0.234) | 0.089 | -2.594(-5.058,-0.130) | 0.040 | -1.652(-3.506, 0.203) | 0.079 |
| Hypertension |  |  |  |  |  |  |  |
| No | Reference | 0.533(-0.482,1.548) | 0.296 | 1.335( 0.060,2.611) | 0.041 | 1.476( 0.421,2.532) | 0.007 |
| Yes | Reference | -1.676(-5.548, 2.196) | 0.388 | -3.128(-6.208,-0.048) | 0.047 | 3.797( 0.613, 6.982) | 0.021 |
| Cardiovascular diseases |  |  |  |  |  |  |  |
| No | Reference | 0.328(-1.040,1.696) | 0.631 | 0.544(-0.633,1.721) | 0.357 | -0.048(-1.142,1.047) | 0.931 |
| Yes | Reference | -0.255(-1.827,1.318) | 0.746 | 1.137(-0.730,3.005) | 0.226 | 5.612( 4.236,6.988) | <0.001 |
| Diabetes mellitus |  |  |  |  |  |  |  |
| No | Reference | 0.338(-0.714,1.389) | 0.521 | 0.993(-0.272,2.259) | 0.121 | 1.693( 0.698,2.688) | 0.001 |
| Yes | Reference | -0.605(-2.635,1.425) | 0.551 | 1.322(-1.006,3.650) | 0.259 | 5.23( 2.854,7.606) | <0.001 |

Notes: Normal sleep was used as reference. Abbreviations: BMI, body mass index.
